# Potential role of intratumor bacteria outside the gastrointestinal tract: More than passengers

**DOI:** 10.1002/cam4.6298

**Published:** 2023-06-28

**Authors:** Zhu Liu, Lian‐Lian Hong, Zhi‐Qiang Ling

**Affiliations:** ^1^ Zhejiang Cancer Institute, Zhejiang Cancer Hospital, Institute of Basic Medicine and Cancer (IBMC), Chinese Academy of Sciences Hangzhou Zhejiang China

**Keywords:** bacterial‐mediated cancer therapy, carcinogenesis, intracellular bacteria, intratumor bacteria, tumor microenvironment

## Abstract

**Introduction:**

Tumor‐associated bacteria and gut microbiota have gained significant attention in recent years due to their potential role in cancer development and therapeutic response. This review aims to discuss the contributions of intratumor bacteria outside the gastrointestinal tract, in addition to exploring the mechanisms, functions, and implications of these bacteria in cancer therapy.

**Methods:**

We reviewed current literature on intratumor bacteria and their impact on tumorigenesis, progression, metastasis, drug resistance, and anti‐tumor immune modulation. Additionally, we examined techniques used to detect intratumor bacteria, precautions necessary when handling low microbial biomass tumor samples, and the recent progress in bacterial manipulation for tumor treatment.

**Results:**

Research indicates that each type of cancer uniquely interacts with its microbiome, and bacteria can be detected even in non‐gastrointestinal tumors with low bacterial abundance. Intracellular bacteria have the potential to regulate tumor cells' biological behavior and contribute to critical aspects of tumor development. Furthermore, bacterial‐based anti‐tumor therapies have shown promising results in cancer treatment.

**Conclusions:**

Understanding the complex interactions between intratumor bacteria and tumor cells could lead to the development of more precise cancer treatment strategies. Further research into non‐gastrointestinal tumor‐associated bacteria is needed to identify new therapeutic approaches and expand our knowledge of the microbiota's role in cancer biology.

## 1 INTRODUCTION

Cancer is caused by genetic changes that perpetuate malignancy.^1^, ^2^ Recent data from investigations of host–microbe interactions suggest that microbiomes, particularly bacteria in tumors, may play a significant role in the acquisition of cancer hallmark capabilities,^3^ bringing the long‐standing question of the relationship between cancer and bacteria back to the forefront.

Historically, attempts to treat cancer with microorganisms were among the early strategies employed to combat the disease. The spontaneous regression of malignancies following bacterial infection has been observed since the 13th century.^4^, ^5^ In the 19th century, Dr. William Coley treated tumors with intratumoral injections of inactivated *Streptococcus* and *Serratia* species and observed a durable regression of the tumors, which was also known as Coley's toxin.^4^, ^6^ It is believed that the effectiveness of Dr. Coley's toxin is related to the activation of the immune system, making him the father of cancer immunotherapy.^6^ The partial success of these bacterial‐based treatments, together with the success of researchers at the time in cultivating bacteria in tumors, led to the hypothesis that malignancies have a bacterial origin.^7^ With the establishment of the somatic mutations theory and the criticism that bacteria cultured from within tumors actually originated from environmental contamination, research on cancer‐related bacteria fell out of favor with academics.

As next‐generation sequencing and bioinformatics technologies advance, however, there is mounting evidence that bacteria may play a significant role in nearly all aspects of cancer hallmarks.^3^, ^8^, ^9^, ^10^ Although most studies of tumor‐associated bacteria have focused on gut microbiota,^11^, ^12^, ^13^ recent advances in this field have broadened the concept of tumor‐associated bacteria to include intratumor bacteria that influence the biological behavior of tumor cells within the microenvironment.^14^, ^15^, ^16^ Limited by the low microbial biomass of non‐gastrointestinal tumor samples, advances in this field began with the aerodigestive tract that has direct contact with the external environment, such as cervical cancer,^17^, ^18^ lung cancer,^19^, ^20^, ^21^, ^22^ skin cancer,^23^, ^24^, ^25^ oral cancer,^26^, ^27^, ^28^ and pancreatic cancer.^29^, ^30^, ^31^ Before the recent availability of improved processing methods for low microbial biomass samples, studies in the field of intratumor bacteria from the aerodigestive tract were deemed unreliable since environmental contamination could not be ruled out.^32^, ^33^, ^34^ To surmount this challenge, principles in order to mitigate the impacts of environmental contamination had been proposed.^35^, ^36^ In combination with the techniques of tissue imaging, 16s RNA sequencing, and culturomics,^37^ there has been robust evidence for the presence of bacteria in tumor tissues, even inside the tumor cells, that are traditionally considered sterile.^14^ Meanwhile, putative cross‐talk mechanisms between intratumor bacteria and tumor cells have also been explored.^14^ Recent research indicates that bacteria within breast cancer cells are capable of metastasizing alongside tumor cells and modulating the cellular architecture of circulating tumor cells, hence promoting their colonization.^38^ Nevertheless, in general, existing studies are confined to presenting intratumor bacteria and comparing them with those of other types of tumor tissues, associated normal tissues, or metastases, and are less concerned with microbial effects on tumor biological behavior or the tumor microenvironment and its underlying mechanisms. This review focuses on the advancements made in the field of intratumor bacteria, particularly those outside the gastrointestinal (GI) tract. The methods used to evaluate intratumor bacteria, the distribution and localization of intratumor bacteria in tumors, the underlying mechanism of crosstalk between intratumor bacteria and tumor cells, and the therapeutic potential and diagnostic value of intratumor bacteria are also discussed in detail.

## 2 PRINCIPLES REQUIRED TO FIND INTRATUMOR BACTERIA

Until recently, the understanding of intratumoral bacteria outside the gastrointestinal tract has been limited, primarily due to challenges arising from the low microbial biomass in samples. Improved sensitivity, however, is a double‐edged sword for samples with low biomass. The main limitations in intratumor bacteria research stem from the similarities between the microbial biomass of tumor samples and their blank controls. This similarity makes it difficult to control environmental contaminants and cross‐contamination, which can disrupt and overwhelm the amplified biological signals. A recent study also reported that contaminant sequences of microbial origin were cataloged as novel human sequences in several human pan‐genome studies.^39^ This issue is particularly prominent in next‐generation sequencing (NGS), which plays a crucial role in intratumor research.^33^, ^34^, ^35^ Every stage of NGS could contribute bias, but DNA or RNA extraction causes the most variability.^40^ Due to the widespread and unavoidable presence of contaminants,^32^ any research in this field, particularly those employing low microbial biomass tumor samples, must employ additional quality control procedures to ensure the reliability of its conclusions. In addition to ensuring sample consistency and representativeness, it is essential to reduce or monitor the influence of contaminant DNA and cross‐contamination during and after the sequencing process. In general, compared to procedures for normal purposes, such as detection of gut microbes, sequencing for low microbial biomass tumor samples necessitates extra care in (i) sampling, (ii) sequencing, and (iii) analyzing to assure reliability.^35^, ^41^ In 2019, Eisenhofer et al. introduced the RIDE checklist (Report, Include, Determine, and Explore) as a minimum guideline to improve the validity of low microbial biomass research.^35^

First, although the introduction of contaminants at the sampling stage is difficult to avoid, modest actions could considerably improve the consistency and comparability of the samples.^35^, ^41^ Different sample groups should be randomized and treated uniformly. Nejman et al. investigated samples from multiple centers, at different periods, and in separate batches, and utilized a particular filter to exclude center‐specific contaminations.^14^

**FIGURE 1:**
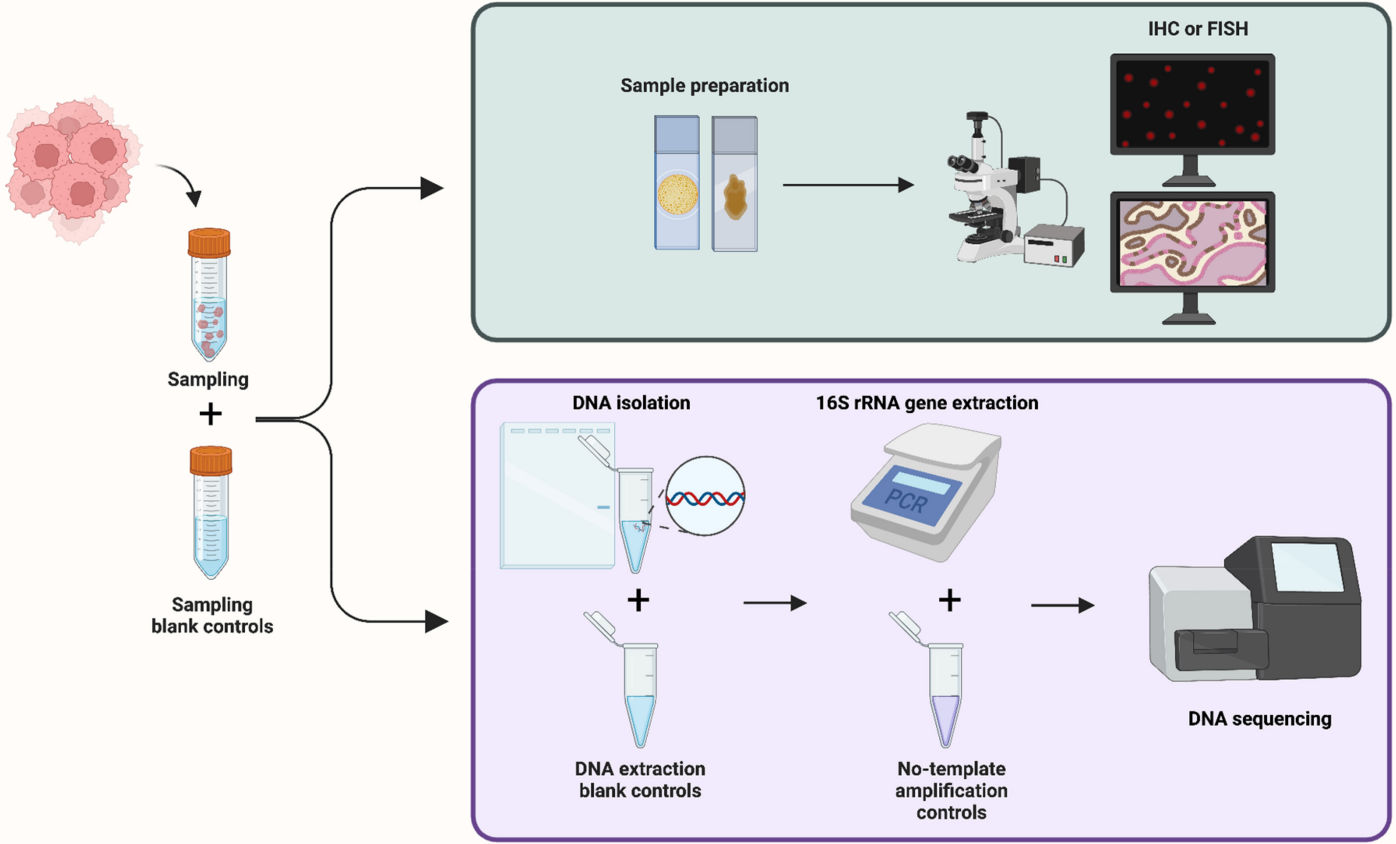
Flowchart of common techniques for detecting and identifying intratumor bacteria, including FISH, IHC, and NGS, as well as the minimum recommended control set‐up to ensure experiment accuracy and reproducibility.

Second, a more intentional configuration of control groups can improve the assay's quality by monitoring contamination. To ensure the reproducibility of the finding, it is advised that all experiments involving low microbial biomass samples should at least be backed up with sampling blank controls, DNA extraction blank controls, and no‐template amplification controls (Figure 1).^35^, ^42^ Depending on the experimental configuration, the control settings must be modified to fulfill the specific experimental purpose. For example, Nejman et al. employed the margins of the paraffin blocks as a control for 16S sequencing utilizing paraffin‐embedded specimens and used the number of control groups of up to 50% of the experimental group to prevent contamination in their PCR procedure.^14^ Optimizing control group settings and experimental protocols increased the sensitivity of intratumor bacteria identification to 1 bacterium per 10,000 tumor cells in a recent study.^38^

Third, the final analysis must accurately interpret the data and eliminate the impacts of contamination. Many relevant studies identify common bacteria discovered in contaminants for cross‐referencing.^32^, ^43^ There is a degree of overlap between the prevalent contaminating species reported in different research, showing that certain bacterial species are more prone to contaminating experimental samples; therefore, such data should be interpreted with caution. However, it is not encouraged to directly exclude the reported microbial species as contamination directly.^43^ Furthermore, the straightforward and conservative approach of excluding all taxa found in negative controls from the analysis is also not recommended, as common contaminating bacteria may also be present in tumor samples, and removing them all could result in the loss of biological signals.^35^ Therefore, we advise more nuanced approaches to data analysis. For example, a sample exclusion value (e.g., *K* 1/2 value) derived based on the positive controls could be used as the cutoff value, the value means approximately 50% of reads will be contaminant.^36^ Similarly, if enough negative controls are included in the experiment, a cutoff value based on the inflection point in the bimodal distribution of the species abundance percentage in control samples can be used to exclude common contaminants.^14^ Bioinformatics reduces contamination after sequencing. Based on the assumption that “the non‐contaminants will appear in a greater proportion of true samples than in negative controls,” the R package Decontam effectively finds and eliminates contaminant DNA sequences, hence enhancing the quality of the study.^44^ Another example is a statistical model developed for removing contaminated species from The Cancer Genome Atlas (TCGA), thereby mitigating batch effects and isolating the tissue‐resident microbiome.^45^

Increased sensitivity to the detection of microorganisms is always accompanied by a greater danger of contamination bias. Due to the impossibility of totally excluding background contamination at the sampling stage, there is an urgent need for improved bioinformatics methods to screen for contaminants and alleviate this dilemma.

## 3 APPROACHES TO FIND INTRATUMOR BACTERIA

It is believed that the composition and function of intratumor bacteria and the biological behavior of tumor cells are interdependent. Various combinations of methods can be utilized to detect or identify these microbial communities, and suitable applications of these toolboxes could aid in our understanding of the oncogenesis and pathogenesis of malignancies.

### 3.1 Next‐generation sequencing

With the completion of the Human Microbiome Project in 2017^46^ and the rapid evolution of microbiological analytical tools such as next‐generation sequencing (NGS), we can amplify and detect weak microbial signals in tumor samples, resulting in a remarkable blossoming of microbiome research.^41^, ^47^, ^48^ NGS does not rely on conventional bacterial culture techniques, allowing it to detect and characterize unculturable intratumor microorganisms. NGS has become a necessary, if not critical, tool for unculturable research and therapeutic applications since a major subset of tumor bacteria are uncultivable.^49^, ^50^ In intratumoral research, the commonly employed next‐generation sequencing (NGS) techniques include 16S rRNA sequencing and metagenomic sequencing.

Amplicon sequencing amplifies a DNA region via PCR before sending it to sequencing is the most common NGS method used for bacterial identification. Most typically, the 16S ribosomal RNA (rRNA) gene is amplified. The 16S rRNA gene exists in almost all bacteria and is responsible for the ribosomal 30S subunit of prokaryotes. 16S rRNA is highly conserved in bacteria but contains highly variable sequence regions (V1–V9) that can distinguish different bacterial species and genera makes it a great marker for identifying bacteria.^51^ However, although sequencing methods based on full‐length 16S rRNA gene have emerged,^52^, ^53^ most 16S rRNA sequencing uses only a subset of the variable regions as amplification targets to limit the cost and time of the sequencing. It is worth noting that since no single hypervariable region can be used to distinguish all bacteria, different choices of hypervariable regions can introduce bias and thus produce different sequencing results.^51^, ^54^, ^55^ Furthermore, two methods are available for further analysis of sequencing results. (i) The distance‐based operational taxonomic units (OTUs) can be used to define a species (with an OTU sequence identity cutoff value of 97%), genus (95%), and phylum (80%) without a reference database at first, and then, taxonomic identification will be performed by aligning to the reference 16S rRNA sequence databases.^49^, ^55^, ^56^ (ii) The non‐distance‐based amplicon sequence variants (ASVs) can be used to directly perform taxonomic identification through exact nucleotide matching dependent on the reference databases.^57^, ^58^, ^59^ Both methods can be employed for estimating taxonomy, and both even have the capability to generate “candidate species” for unculturable microbes. Moreover, there is still a debate about which approach performs better for tumor‐associated bacteria analysis,^60^ and in the field of intratumor bacteria studies, we need more data to show whether the choice of different methodologies affects the accuracy of the study using low microbial biomass samples.

Unlike amplicon sequencing, metagenomic sequencing is based on all the DNA in a given sample. The DNA is fragmented, and the reads are aligned to a reference database. With adequate sequencing depth, metagenomic sequencing is capable of analyzing whole microbial genomes based on the short DNA sequence reads.^51^ Therefore, metagenomic sequencing theoretically provides more information about the bacterial function and biological behavior, and has the ability to detect previously unknown microbes.^61^ Compared to the 16S rRNA sequencing, metagenomic sequencing could offer a higher resolution and sensitivity for differences that occurred on the species level, and have the ability to detect strain‐level changes, which is difficult with 16S rRNA sequencing.^61^, ^62^, ^63^ The main obstacle to the widespread use of metagenomic sequencing is cost and contamination during sampling; much more sequence data are required for metagenomic sequencing compared with 16S rRNA sequencing.^51^ In 2021, using metagenomic sequencing, researchers revealed the tumor‐suppressing effect of *Lactobacillus gallinarum* in human colorectal cancer (CRC).^64^ In combination with metagenomic sequencing and 16S rRNA sequencing, Matson et al. reported a significant association between commensal microbial composition and immune checkpoint inhibitor response.^65^

### 3.2 Methods to intratumor bacteria detection

NGS techniques have the advantage of high sensitivity and throughput in intratumor bacteria studies. However, it cannot provide information on microorganism ecology and distribution. Fluorescent in situ hybridization (FISH) is a method that can monitor and identify different individual microorganisms in pathology samples, which is especially advantageous for uncultivable microorganisms. FISH targets relatively stable rRNA (16S and 23S) and uses probes highly complementary to target sequences to detect and quantify bacteria in samples.^66^ It is widely used in intratumor bacteria related studies.^14^, ^30^, ^31^, ^38^, ^67^ Recently, in a study of intracellular bacteria, FISH‐based subcellular localization revealed the presence of bacteria at the perinuclear region.^38^ Similarly, traditional approaches that base antigen–antibody binding such as immunohistochemistry (IHC) or immunofluorescence (IF) to detect bacteria proteins also play an important role in this area.^14^, ^38^ High‐resolution electron microscopy (EM) is another widely used approach to directly observe and identify intratumor bacteria.^38^, ^68^, ^69^ The main shortcoming of EM is the grayscale images of ultrastructure information make molecules hard to recognize. To solve this problem, correlative light and electron microscopy (CLEM) combined the strengths of EM and fluorescence microscopy, enabling researchers to identify specific molecules within its ultrastructural context.^70^ In the intratumor bacteria field, Kalaora et al. demonstrated the bacteria (*Fusobacterium nucleatum*, *Actinomyces odontolyticus*, and *Staphylococcus caprae*) entered the melanoma cells with CLEM.^71^ Analogously, CLEM clearly demonstrated the intracellular localization of bacteria in four tumor types (breast cancer, lung cancer, melanoma, and glioblastoma).^14^

### 3.3 Culture of intratumor bacteria

The advent of NGS has enabled more comprehensive exploration and improved understanding of the human microbiome, generating vast amounts of data on tumor‐related microbiota, primarily through amplicon sequencing and whole‐genome metagenomics. Nonetheless, NGS has several limitations, including the potential to overlook minority species, insufficient information on viability, and limited availability of strains for further experimentation.^37^ These limitations make cultivation an indispensable complement for describing novel microbial species and an powerful approach which significantly enhances our understanding of microbial diversity.^72^ Although the majority of human bacteria are difficult to isolate and considered unculturable,^73^ advancements in microbial culture techniques, such as membrane diffusion‐based cultivation and microfluidic systems, have enabled scholars to successfully isolate and identify culturable bacteria within various tumors, including colon cancer,^74^ pancreas cancer,^31^ lung cancer,^20^ and breast cancer.^75^ The development of microbial culture techniques led to the emergence of culturomics, an approach that employs multiple culture conditions, matrix‐assisted laser desorption/ionization time‐of‐flight (MALDI‐TOF) mass spectrometry, and 16S rRNA sequencing for the identification of bacterial species.^37^ Recently, a research utilize culturomics to explore the pulmonary and oral microbiota alterations in patients with lung cancer. The researchers successfully isolated and identified low abundance, yet functionally significant bacteria from bronchoalveolar lavage fluid (BALF) samples which may have been overlooked by traditional sequencing methods. The findings revealed shifts in the microbial community composition of lung cancer patients, with potential site and pathology‐dependent variations.^76^ Furthermore, as a powerful tool for elucidating bacterial function, culturomics has become a key player in the discovery of anti‐cancer probiotics and other preventive or therapeutic fields. Researchers have identified several strains associated with the efficacy of immune checkpoint inhibitor treatment, including Bifidobacterium species (*B. longum*, *B. breve*, *B. adolescentis*), Enterococcus hirae, Bacteroides fragilis, Bacteroides thetaiotaomicron, Barnesiella intestinihominis, and Akkermansia muciniphila.^13^, ^77^, ^78^, ^79^ Altogether, the application of culturomics have the potential to broaden the scope of intratumor bacteria research and advance the development of cancer therapeutic strategies. Nevertheless, due to the low biomass of intratumor microbes, cultivating intratumor bacteria outside the gastrointestinal tract remains challenging, resulting in fewer studies in this area. However, with advancements in microbial culture techniques and culturomics, research efforts in this crucial field are progressively gaining attention, and further progress is anticipated.

## 4 THE ROLE OF INTRATUMOR BACTERIA IN TUMOR PROGRESSION

**FIGURE 2:**
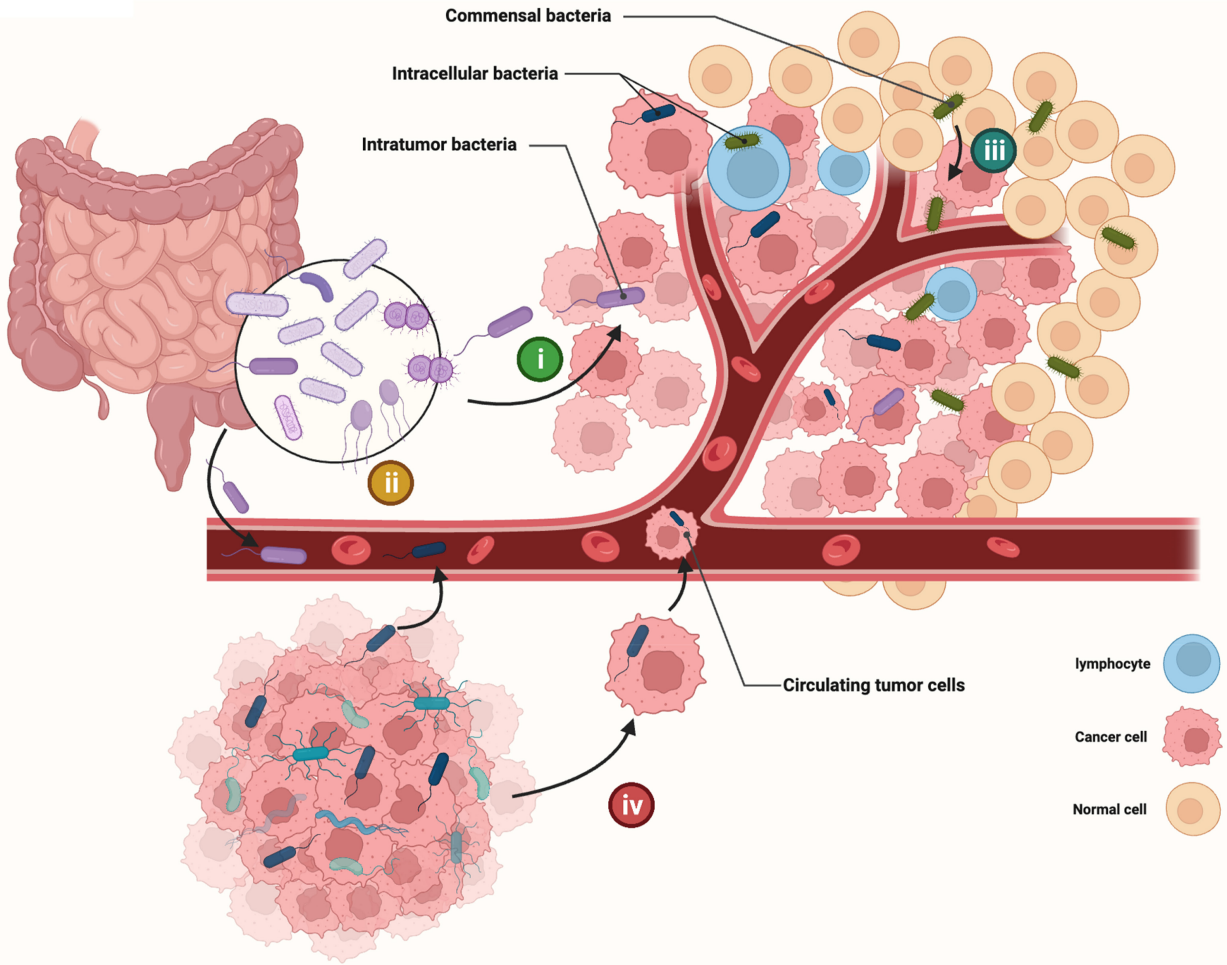
Four possible origins of bacteria in tumors outside the gastrointestinal tract: (i) Migration or invasion from the gastrointestinal tract; (ii) transfer from the circulatory system; (iii) transfer from the cardiovascular system; (iv) carried through a metastatic process by circulating tumor cells.

Unlike gastrointestinal tumors, where the gut microbes are their intratumor microbes, the sources of bacteria in tumors originating outside the gastrointestinal tract are not yet clearly understood, and the composition and abundance of intratumor bacteria vary significantly between cancer types (Table 1). What we can anticipate is that tumors in different organs have different bacterial origins.^80^ Generally speaking, bacteria in tumors outside the gastrointestinal tract have four possible origins (Figure 2). (i) Migrated or invaded from the gastrointestinal tract. Pancreatic cancer possesses a similar bacterial profile to the duodenum, and the pancreatic ducts are likely to be a conduit for retrograde migration of bacteria.^16^, ^30^, ^31^ (ii) Transferred from the circulatory system, such as oral *F. nucleatum* might probably colonize breast cancer via the hematogenous route mediated by Gal‐GalNAc expression.^81^ (iii) Selective colonization originating from normal adjacent tissues. In many tumor types, tumor tissue shares a highly similar bacterial profile with its adjacent normal tissue.^14^, ^19^, ^82^ (iv) Carried by circulating tumor cells through the process of metastasis. For example, intratumor bacteria of breast cancer can invade into tumor cells, colonizing the lungs along with the metastasis of tumor cells.^38^

**TABLE 1:** Intratumor bacteria in different cancers.

Tumor type	Microorganisms	Localization	Changes	Function	Raf
Bladder cancer	*Barnesiella, Parabacteroides, Prevotella, Alistipes, Lachnospiracea_incertae_sedis, Staphylococcus*	In tumor tissues	Increase		^145^
Breast cancer	*Streptococcus, Lactobacillus, Staphylococcus, Enterococcus*	In tumor cells	Increase	Enhancing resistance to fluid shear stress by reorganizing Actin cytoskeleton through Rhoa‐ROCK pathway	^38^
Breast cancer	*Fusobacterium nucleatum*	In tumor tissues	Increase	Suppressing accumulation of tumor‐infiltrating T cells and promotes tumor growth and metastatic progression	^81^
Breast cancer	*Fusobacterium, Atopobium, Gluconacetobacter, Hydrogenophaga, Lactobacillus*	In tumor tissues	Increase	Related to carcinogenesis	^146^
Breast cancer	*Bacteroides fragilis*	In tumor tissues	Increase	Inducing growth and metastatic progression of tumor cells through‐catenin and Notch1 axis	^90^
Breast cancer	*Porphyromonas, Lacibacter, Ezakiella, Fusobacterium*	In tumor tissues	Increase	Related to higher stage tumors	^147^
Breast cancer	*Alkanindiges, Micrococcus, Caulobacter, Proteus, Brevibacillus, Kocuria, Parasediminibacterium*	In tumor tissues	Decrease	Related to ER positive tumors	^147^
Breast cancer	*Pelomonas, Ralstonia, Oblitimonas, Lactobacillus, Methylophilus, Achromobacter*	In tumor tissues	Increase	Related to PR positive tumors	^147^
Breast cancer	*Cloacibacterium, PRD01a011B, Alloprevotella, Stakelama, Filibacter, Blastomonas, Anaerostipes*	In tumor tissues	Increase	Related to HER2 positive tumors	^147^
Breast cancer	*Azomonas*	In tumor tissues	Increase	Related to triple‐negative breast cancer (TNBC) tumors	^147^
Endometrial cancer	*Pseudoramibacter_Eubacterium, Rhodobacter, Vogesella, Bilophila, Rheinheimera, Megamonas*	In tumor tissues	Decrease		^148^
Endometrial cancer	*Pelomonas, Prevotella*	In tumor tissues	Increase	Related to tumor burden	^149^
Hepatocellular carcinoma	*Stenotrophomonas maltophilia*	In tumor tissues	Increase	Promoting carcinogenesis by activating the senescence associated secretory phenotype (SASP)	^91^
Lung cancer	*Herbaspirillum, Sphingomonadaceae*	In tumor tissues	Increase	Promote Lung Cancer Development via γδ T Cells	^20^
Lung cancer	*Acidovorax*	In tumor tissues	Increase	Related to squamous cell carcinoma with TP53 mutation	^89^
Lung cancer	*Lactobacillus rhamnosus*	In tumor tissues		Promoting immunity against B16 lung metastases	^92^
Lung cancer	*Veillonella, Rothia*	In tumor tissues	Increase	Related to metastasis	^93^
Lung cancer	*Streptococcus*	In tumor tissues	Decrease	Related to metastasis	^93^
Lung cancer	*Veillonella parvula*	In tumor tissues		Related to worse prognosis mediated by IL‐17 producing and PI3K, MAPK, and ERK activating	^19^
Melanoma	*Fusobacterium nucleatum, Shewanella dokdonensis*	In tumor cells		Peptides be presented by tumor cells and elicit immune reactivity	^71^
Nasopharyngeal carcinoma	*Prevotella, Porphyromonas*	In tumor tissues	Increase	Related to relapse	^100^
Ovarian cancer	*Aquificae, Planctomycetes*	In tumor tissues	Increase		^150^
Ovarian cancer	*Acinetobacter*	In tumor tissues	Increase	Related to better prognosis	^151^
Pancreatic cancer	*Bifidobacterium pseudolongum*	In tumor tissues	Increase	Inducing suppressive monocytic cellular differentiation via TLR signaling to induce immune tolerance	^30^
Pancreatic cancer	*Gammaproteobacteria*	In tumor tissues		Inducing chemoresistance by metabolizing chemotherapeutic agents into its inactive forms	^16^
Pancreatic cancer	*Pseudoxanthomonas, Streptomyces, Saccharopolyspora, Bacillus clausii*	In tumor tissues		Related to better prognosis	^31^
Prostate Cancer	*Escherichia coli, Propionibacterium acne, Staphylococcus, Chlamydia trachomatis*	In tumor tissues		Related to the carcinogenesis	^152^
Thymoma	*Sphingomonas, Phenylobacterium*	In tumor tissues	Increase		^153^
Thyroid cancer	*Sphingomonas, Aeromonas*	In tumor tissues	Increase	Related to poor prognosis	^154^

In addition to the source of intratumor bacteria, the tumor itself provides unique conditions for bacterial colonization.^41^ (i) The aberrant or dysfunctional tumor neovascularization allows circulating bacteria to embed in tumor tissue. (ii) The suppressive immune microenvironment in tumors leads to easier survival of bacteria. (iii) Hypoxic region in solid tumors provide suitable environments for anaerobic bacteria. (iv) Necrotic regions within the tumor provide sufficient nutrients to attract bacterial colonization. A consequence of these distinct characteristics is that tumor tissue tends to have a higher abundance of bacteria than normal tissue.^16^, ^71^, ^83^ However, several investigations have found that tumor tissues exhibit a higher bacterial burden but less diversity.^20^, ^38^ Evidence is mounting that intratumor bacteria are involved in multiple steps of tumor initiation, development, and progression. It is necessary to analyze the underlying mechanism of tumor‐bacteria crosstalk in different cancers and identify their roles in the progression of cancers.

### 4.1 Tumorigenesis

Genome instability and mutations are one of the enabling characteristics of cancer.^3^ Among the many causes that can lead to genetic alterations, DNA damage caused by intratumor bacteria has been widely reported recently. However, most of the current reports in this field focus on GI tract tumors which the Gut microbes are their intratumoral bacteria.^84^, ^85^, ^86^, ^87^ Outside the GI tract, to date there is little agreement on whether intratumor bacteria may directly cause carcinogenesis. However, there is a consensus that the microbiome can take advantage of environmental factors and epigenetically/genetically destabilize cells to exert carcinogenic effects.^88^ A notable example is that bacteria in lung adenocarcinoma (LUAD) can promote tumorigenesis in mouse models driven by an activating point mutation of KRAS and loss of TP53 through γδT cells. In addition, another study reported that in lung squamous cell carcinoma (LSCC), *Acidovorax* is enriched in smokers and exhibits a higher abundance in cases with TP53 mutations, indicating a microbiome‐gene interaction that may contribute to tumorigenesis.^89^

In addition to the direct or indirect effects on genome stability, the influence of bacteria on oncogenic pathways serves as another mechanism of carcinogenesis.^3^ Researchers have reported that enterotoxigenic *Bacteroides fragilis* (ETBF) present in breast cancer can stimulate epithelial hyperplasia in the mammary gland through *B. fragilis* toxin (BFT) secretion. Cancer cells can even form a long‐term oncogenic memory from the initial exposure to BFT, which could promote the migration, invasion, and stemness‐rich phenotype formation through β‐catenin and Notch1 pathways.^90^ Liu et al. revealed that an opportunistic pathogen—*Stenotrophomonas maltophilia*, is enriched in hepatocellular carcinoma (HCC) tissues, which could drive the progression of liver cirrhosis toward hepatocellular carcinoma and promote carcinogenesis by activating the senescence associated secretory phenotype (SASP) in hepatic stellate cells (HSCs) to secrete IL‐1β through the TLR4/NF‐κB/NLRP3 pathway.^91^ Similar to the relationship between genetic alterations and intratumor bacteria, most current studies focus on GI tumors, while many unsolved questions about the effect of intratumor microbiota on oncogenic pathways in non‐GI cancers still need to be addressed.

### 4.2 Metastasis

**FIGURE 3:**
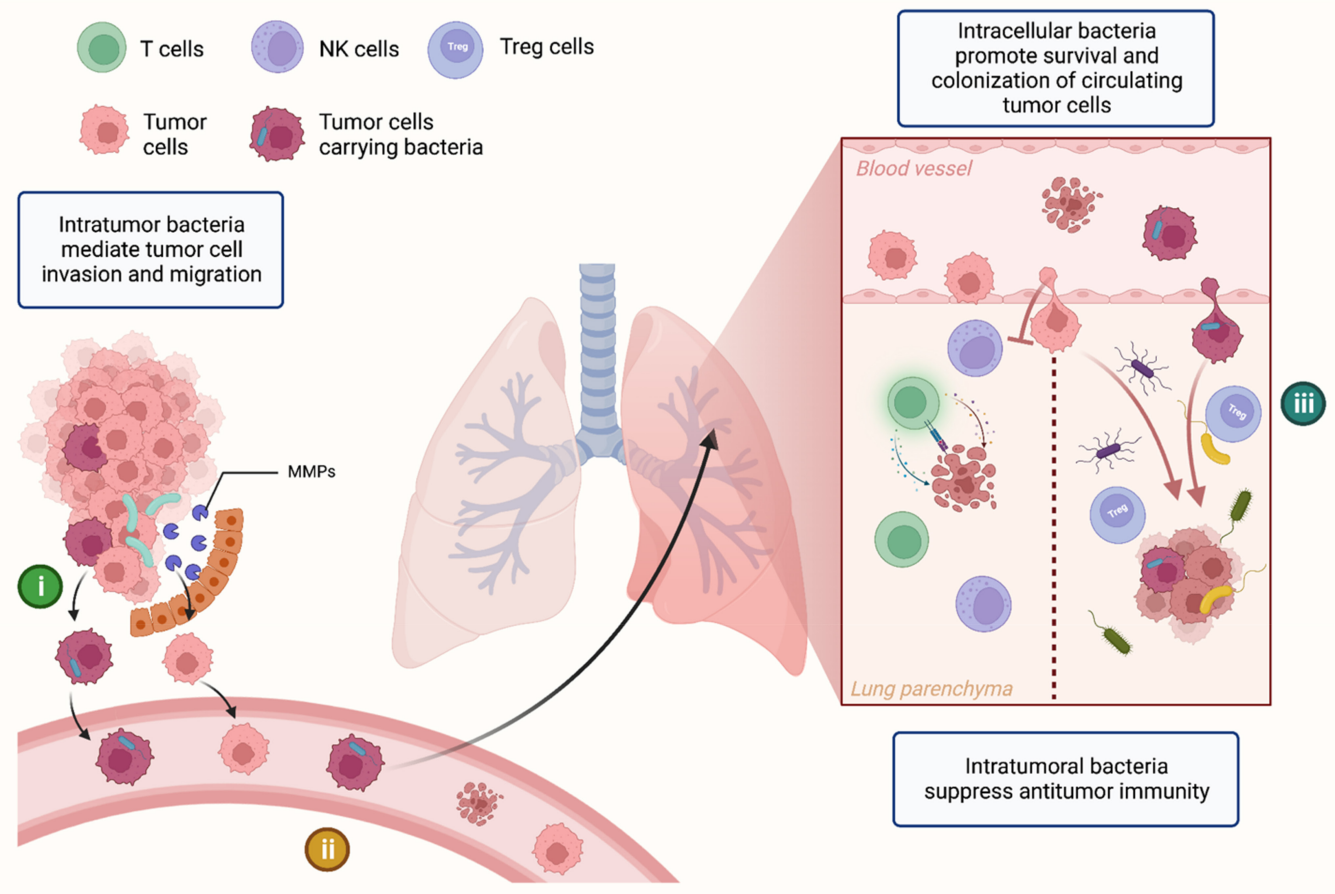
Three stages of tumor metastasis are facilitated by intracellular bacteria: (i) invasion and migration, (ii) maintenance of circulating tumor cell survival, and (iii) targeted colonization of tumor cells. Both in the primary tumor and in the metastases, intratumor bacteria are crucial to the metastatic process.

Intratumor bacteria are involved in all stages of tumorigenesis, development, and metastasis. In addition to bacteria from the primary tumor site, commensal bacteria from the metastatic site, and even the bacteria inside the circulating tumor cells are also involved in the process of metastasis and colonization (Figure 3). In a mouse model, bacterial load in lung tissue is connected to melanoma B16 lung metastases mediated by Treg cell, T cell, and NK cell activity.^92^ Antibiotics can inhibit this pro‐metastatic effect, indicating that commensal bacteria in normal tissue form a milieu permissive to metastasis. Parhi et al. reported that *Fusobacterium nucleatum* in breast cancer could induce the expression of matrix metalloproteinase 9 (MMP9) and mediate the metastasis of breast cancer.^81^ Intratumor bacteria can also promote tumor cell invasion and migration, according to several studies.^92^, ^93^ Recently, Fu et al. have revealed that bacteria can even play a pro‐metastatic role inside circulating tumor cells that enter the circulatory system during metastasis.^38^ In this study, using the murine spontaneous breast‐tumor model MMTV‐PyMT, the authors confirmed that intratumor bacteria invaded the tumor cells in the primary tumor site, and become intracellular bacteria, enhancing resistance to fluid shear stress by reorganizing the actin cytoskeleton of tumor cells during the process of metastatic colonization. As we know, metastasis is a rather inefficient process,^94^ with many tumor cells exiting primary tumors and entering the blood circulation, only a very few of them eventually result in metastatic colonies. Findings by Fu et al. partially address this question. In terms of metastasis, bacteria are involved in all three aspects of (i) the promotion of invasion and migration, (ii) the maintenance of circulating tumor cells survival, and (iii) the selective colonization of tumor cells. Therefore, further research should be undertaken to investigate the underlying mechanisms of the bacteria‐metastasis relationship, which might be a potential target for cancer treatment.

### 4.3 Immune microenvironment

Gut bacteria can alter the anti‐cancer immune microenvironment of tumors arising from the GI tract (e.g. colorectal cancer),^95^, ^96^, ^97^ or from distant organs (e.g. breast cancer or lung cancer).^31^, ^98^, ^99^ Recently, researchers broadened this field to non‐GI intratumor bacteria. Researchers have found that intratumor bacteria are able to influence both tumor infiltration and the function of immune cells. The diversity of intratumor bacteria was found to influence both the extent of immune infiltration and the degree of activation of CD8^+^ T cells.^31^ In addition, consistent with the observation in colorectal cancer, the tissue load of *F. nucleatum* in breast cancer was found adversely linked with infiltrating lymphocytes.^81^ A negative correlation was also found between intratumor bacterial load and T‐lymphocyte infiltration in nasopharyngeal carcinoma (NPC).^100^ Conversely, depending on the phylum and genus, bacteria inoculation in tumors has also been found to increase tumor‐infiltrating lymphocytes, exerting an immunostimulatory effect.

In addition to modifications in immune cell infiltration, exhaustion of immune cells due to suppressive immune microenvironment and immune reprogramming is also thought to be an essential reason for tumor immune evasion. Intratumor bacteria were linked to immunogenic reprogramming in pancreatic ductal adenocarcinoma (PDA), including an increase in myeloid‐derived suppressor cells and a decrease in M1 macrophage differentiation.^30^ Bacterial ablation can enhance CD4^+^ T‐cell TH1 differentiation and CD8^+^ T‐cell activation, along with PD‐L1 upregulation.^30^ The authors also reported that the tolerogenic immune program generated by intratumor bacteria is the result of the activation of Toll‐like receptors in monocytic cells.^30^ Le Noci and colleagues observed that lung bacteria promote melanoma lung metastasis by increasing Treg cells and decreasing NK cell activation, leading to a immunosuppressive microenvironment.^92^ Intratumor bacteria stimulated production of IL‐1β and IL‐23, activating Vg6^+^ Vd1^+^ gd T cells to produce IL‐17 and generating a pro‐tumor inflammatory microenvironment.^20^ Further, Tsay et al. found that dysbiosis of *Veillonella parvula* causes IL‐17 inflammatory phenotype, checkpoint inhibitor activation, higher tumor burden, and worse lung cancer prognosis. Besides IL‐17, PI3K, MAPK, and ERK pathways may also contribute to this process.^19^ Nejman et al. identified bacteria inside CD45^+^ immune cells, which may alter or reflect the tumor's immunological status. Furthermore, specific bacteria taxa differences have been found through the comparison between the immune checkpoint inhibitors (ICI) responders and nonresponders.^14^ However, the exact effects and the underlying mechanisms of intratumor bacteria on cancer cells and the immune system remain unknown. Considering the application of ICI treatment in various types of cancer, understanding these effects is crucial for future therapy possibilities.

## 5 THE ROLE OF INTRACELLULAR BACTERIA

As our understanding of bacteria and tumors evolves, information from intratumor bacteria has given us a microscopic perspective. In a milestone study conducted in 2020, researchers analyzed 1526 tumors along with their adjacent normal tissues across seven different cancer types, and found that each tumor type harbors a unique microbiome composition, with intratumor bacteria predominantly residing within cells. With the application of transmission electron microscopy (TEM) and CLEM, the authors demonstrate that intratumor bacteria are able to invade and persist in the cytoplasm of tumor or immune cells.^14^ The same study discovered intracellular bacteria maintain a cell wall–deficient condition, like L‐forms. To our knowledge, such cell wall‐deficient bacteria can avoid drugs that target the cell wall, and the lack of pathogen‐associated pattern‐recognition receptors makes L‐form bacteria challenging to identify by the immune system.^101^, ^102^ Moreover, the absence of the cell wall barrier makes it easier for this L‐form like bacteria to exchange DNA with the environment. Given their intracellular localization in tumor cells, the relationship between intracellular bacteria and tumor cells deserves further investigation.

In 2022, Fu et al. introduce a study that push this area a step further, revealing that intracellular bacteria may have a profound effect on the biological behavior of tumor cells. In breast cancer, about 80% of intratumor bacteria are present in the form of intracellular bacteria and have a pro‐metastasis function.^38^ Interestingly, this pro‐metastasis effect of intracellular bacteria on breast cancer cells does not seem to depend on the biological function of bacteria, but on the ability of the bacteria to invade the cytoplasm. The very act of entering the cell can lead to cytoskeletal alterations and promote the survival and colonization of circulating tumor cells. Another study in melanoma indicated that intratumor bacteria can infiltrate melanoma cells by analyzing the bacteria's HLA peptides. The authors have found that bacteria peptides can be presented by the HLA‐I and HLA‐II molecules of melanoma tumor cell.^71^ Therefore, intracellular bacteria in melanoma are expected ultimately to modulate immune function. Further, these non‐self‐antigens have the potential to be used as targets for immunotherapy. Bacteria in pancreatic cancer can cause gemcitabine resistance in tumor cells, and immunohistochemistry with lipopolysaccharides (LPS) labeling demonstrates the bacteria are inside the cancer cells.^16^ Therefore, intracellular bacteria are a key component of intratumor bacteria and may promote tumor development. Given that most of intratumor bacteria are indeed intracellular in certain cancers, we may need to rethink the interactions between tumor, bacteria, and tumor microenvironment (TME). However, as investigations in this field are sparse, it is necessary to analyze the intracellular bacteria in different cancers and understand their roles in the progression of cancers.

## 6 THE ROLE OF INTRATUMOR BACTERIA IN ANTITUMOR TREATMENTS

With the deepening understanding that the intratumor bacteria may impact or reflect cancer development, utilization of bacteria‐based personalized data to increase the accuracy of diagnosis and improve prognosis constitutes a promising treatment strategy, providing critical insight for the development of new therapies.

### 6.1 Biomarkers for diagnosis and prognosis

Although the actual cause‐and‐effect link between intratumor bacteria and cancers is unclear, their correlation is well‐established.^42^, ^80^ Poore et al. reported in 2020 that blood‐based microbial DNA (mbDNA) could be utilized as a diagnostic tool to distinguish patients with multiple types of cancer (prostate, lung, and melanoma) and cancer‐free healthy individuals based on distinct microbial signatures. The authors screened their bacteria data using a very stringent decontamination filter that discarded up to 92.3% of total sequence data to ensure the accuracy of the final result.^15^ The microbial signatures remained predictive in applying to patients with low‐grade tumor stages (Ia–IIc). It suggests that the tumor‐associated microbiome is sufficiently altered even at the early stages of tumor development. These microbial profiles may be useful for early detection of cancer and can help with decisions in dilemmas associated with the watch and wait (WW) strategy and anti‐cancer intervention. Similarly, microbiome signatures in prostate cancer corresponded with grades, stages, and scores, could aid in clinical diagnosis.^83^

In terms of prognosis, a study regarding pancreatic adenocarcinoma (PDAC) analyzed the composition of intratumor bacteria in PDAC patients with short‐term survival (STS) and long‐term survival (LTS), generating an intratumoral microbiome signature (*Pseudoxanthomonas–Streptomyces–Saccharopolyspora–Bacillus clausii*) which could be highly predictive of long‐term survivorship.^31^ Lower airway microbial profiles are associated with lung cancer prognosis. *Veillonella parvula* dominates this association.^19^ Furthermore, researchers have analyzed the intratumor bacterial species with various clinical variables, discovering that the increased abundance of most bacteria in the liver was associated with a poorer prognosis for HCC patients. Alcohol or hepatitis B virus (HBV) exposure might provide opportunities for intratumor bacteria to promote HCC development.^103^ In a retrospective cohort study, high bacteria load in nasopharyngeal carcinoma (NPC) corresponded to a poorer prognosis. Intratumor bacteria load could be a robust prognostic tool in NPC patients.^100^ Altogether, these findings support the notion that with proper sampling and appropriate data analysis, the intratumor bacteria can serve as an adequate biomarker for diagnosis and prognosis.

### 6.2 Intratumor bacteria and anti‐cancer therapies

As a component of the tumor microenvironment, increasing attention has been focused on improving our understanding of the relationship between intratumor bacteria and anti‐cancer therapies. Gut microbiota research has revealed the role bacteria may play in cancer treatment, leading us to wonder if intratumor bacteria outside the GI tract could also influence cancer treatment. Recently, researchers have tried various approaches to modulate, even reprogram bacteria for tumor treatment or for its use in combination with other antitumor therapies. In this section, we will discuss the specific influence of intratumor bacteria on tumor treatments and its underlying mechanisms.

As evidence suggests that intratumor bacteria play an active role in tumor development, a fairly straightforward approach would be to use antibiotics to eliminate these unwanted bacteria. However, bacteria in the human body form a highly complex functional network, and intratumor bacteria and gut microbes may not act in concert regarding cancer, or even contradict each other. Furthermore, the broad‐spectrum antibiotics which been broadly used in clinical environment could bring unexpected effects in diversity of the patient's normal microbiota, which could generate misleading results.^16^, ^38^, ^74^ In this case, a selective approach to bacteria elimination is particularly important. For selection against intratumor bacteria, Linda et al. used the antibiotic cocktail (ATBx) consisting of vancomycin, imipenem, and neomycin to eliminated gastrointestinal but not skin bacterial load, and revealed that commensal bacteria could impair the response of immunotherapy and chemotherapy.^104^ Fu et al. have found that administration of ATBx through drinking water (DW) can eliminate both gut and intratumor bacteria of breast cancer, while intravenous (IV) injection could eliminate intratumor bacteria while leaving the gut microbiota intact.^38^ In the same research study, the authors also reported that the intracellular bacteria can survived under administration of impermeable antibiotics (e.g., Ampicillin and Gentamicin), but were eliminated under penetrating antibiotics treatment (e.g., doxycycline). Metronidazole treatment resulted in the reduction of local bacterial burden in lung cancer without substantially reducing the overall gut bacteria.^20^ These results provide us with tools to distinguish the function of gut and intratumor bacteria, and enable us to have the opportunities to manipulate them under certain therapies. Nonetheless, evidence for the application of selective antibiotics in other tumor types is still lacking, and future studies should discreetly address the discrepancy between intratumor and gut bacteria when using antibiotics.

The evidence of the application of antibiotics to interfere with intratumor bacteria in tumor treatment is conflicting. Application of ciprofloxacin eliminates bacterial‐mediated gemcitabine chemoresistance in pancreatic cancer, through a mechanism of eliminating the bacteria which metabolize the gemcitabine (2′,2′‐difluorodeoxycytidine) into its inactive form (2′,2′‐difluorodeoxyuridine), which was reported in colon cancer previously.^16^ In another study, ancomycin/neomycin reduced melanoma B16 lung metastases.^92^ Jin et al. have found that metronidazole administration reduces tumor growth in mouse models with KRAS mutation and TP53 loss.^20^ In lung and pancreatic cancer, antibiotics can convert an immune‐tolerant TME into an immune‐activated one.^30^, ^92^ However, several studies suggest that the administration of antibiotics decreases patient survival. For instance, in a prospective, multicenter, cohort study including patients of non–small cell lung cancer, melanoma, and other tumor types, administration of broad‐spectrum antibiotics prior to immune checkpoint inhibitors (ICIs) treatment was associated with worse overall survival (OS) and higher disease refractory rate.^105^ In addition, studies in patients with renal cancer, non‐small cell lung cancer (NSCLC), or bladder cancer showed that antibiotics were associated with reduced clinical benefit from ICI.^13^, ^106^ Most of the evidence of antibiotics administration that decreases patient prognosis was based on the effect of broad‐spectrum antibiotics on gut bacteria, emphasizing that careful attention should be paid when using antibiotics in cancer patients. Furthermore, to date in the intratumor bacteria field, most studies are retrospective, which have limitations such as the inability to distinguish between the effects of gut bacteria and actual intratumor bacteria, using animal models can get around this to some extent, as well as more prospective evidence is needed to investigate whether antibiotic‐targeted eradication of intratumor bacteria can benefit tumor therapy.

### 6.3 Weaponized bacteria as cancer therapy

In recent decades, bacteria‐based cancer therapy, which involves utilizing live bacteria with inherent tumor‐targeting and tumor‐killing activities to selectively colonize and combat tumors, has been considered a promising approach to overcome cancer. However, early attempts often encountered challenges such as septic shock and infectious death, which limited the progress of this therapeutic strategy.^107^ With the development of genetic editing technology, we can attenuate the virulence of bacteria, as well as improve its anti‐cancer efficiency through manual intervention.^108^ Researchers have modified *Salmonella*, *Listeria*, and *Clostridium* to boost the tumor‐targeting capabilities of obligate anaerobic bacteria and minimize the systemic toxicity of facultative anaerobic bacteria.^109^ Therefore, attenuated, low‐toxic bacteria are increasingly being employed as anti‐cancer medications or therapeutic platforms. In fact, the relationship between intratumor bacteria and bacteria‐based therapies is intimated. Whether by invasion of bacteria due to the nutrition and oxygen level in the tumor or passive entrapment in the aberrant leaky tumor vasculature, the mechanism which intratumor bacteria take advantage of the tumor environment can also be the mechanism by which bacterial‐based therapy can be effective.^107^ These weaponized bacteria can become a promising strategy for anti‐cancer therapies.

#### 6.3.1 Bacterial as drugs

The use of bacteria to treat malignant tumors was one of the first human attempts at tumor treatment and was highly controversial but has regained attention. Researchers are interested in how bacteria infiltrate tumors.^110^ For instance, *Salmonella enterica serovar Typhimurium* has been shown to accumulate in tumors, and have inherent anti‐cancer activities, anti‐angiogenesis activity, and immune‐activating activity, and can be easily genetically manipulated.^111^, ^112^, ^113^, ^114^ VNP20009, a famous genetically engineered *S. Typhimurium* strain, was created by deleting *msbB* and *purI*, resulting in decreased virulence due to dysfunction of LPS and the adenine metabolic pathway.^115^ VNP20009 increases caspase‐3 activity and up‐regulates Bax to cause cancer cell apoptosis.^116^ Despite clinical trials demonstrated that VNP20009 shows no significant antitumor effect in cancer patients,^117^, ^118^, ^119^ there are still various attenuated *Salmonella* strains in addition to VNP20009 (e.g., YB1, A1‐R, and SL7207) that are being extensively investigated given their excellent properties for use in anti‐cancer therapies.^120^, ^121^, ^122^ In addition to *S. Typhimurium*, genetically engineered *Listeria* could kill cancer cells directly by activating NADPH and raising intracellular Ca^2+^.^123^ Furthermore, a *Listeria* strain was genetically modified to target HER2‐expressing tumor cells utilizing biotin‐streptavidin binding.^124^ *Clostridium* secretes various exotoxins, which could directly kill cancer cells. As a promising tool to fight cancer, intratumorally injected attenuated Clostridium novyi‐NT (C. novyi‐NT), resulted in tumor cell lysis and activated inflammation, which was confirmed in a clinical trial though the toxic side effects were significant.^125^

In addition to directly killing the tumor cells, activation of effective anti‐cancer therapy is another approach to bacteria‐based anti‐cancer therapy.^119^, ^126^ Attenuated bacteria can activate the inflammation pathway, for example, attenuated *Salmonella* can elicit a strong cytokine and chemokine storm which result in an increase in tumor‐infiltrating lymphocytes. Then, lysed tumor cells release ATP, which further increases the cytokine and chemokine storm and leads to tumor‐infiltrating lymphocytes activation.^127^ The activation of immunostimulatory factors (e.g., IL‐1β, TNF‐α, and IFN‐γ) and inhibition of immunosuppressive factors (e.g., Arg‐1, IL‐4, and TGF‐β) were observed during the bacteria‐based anti‐cancer therapy.^128^, ^129^, ^130^, ^131^, ^132^ Attenuated bacteria can be recognized by pathogen‐associated molecular patterns (PAMPs), activating the TLRs and resulting in a stimulated immune microenvironment.^133^ Therefore, various attenuated bacterial strains such as *Listeria monocytogenes, Salmonella enterica serovar typhimurium*, and *Escherichia coli* are employed as cancer vaccines.^133^ The modulation of the inflammation pathway leads to a transformation of immune related cells, including T cells,^131^, ^134^ NK cells,^113^ neutrophils, and macrophages.^114^, ^128^

#### 6.3.2 Bacterial as platforms

Inspired by the advantages of bacteria‐based anti‐cancer therapies, researchers have made genetically engineered bacteria platforms to deliver various anti‐cancer agents. Instead of directly killing, these approaches enable bacteria to be a vehicle that releases their payloads in tumor tissues during colonization.^135^ An intriguing approach takes advantage of the characteristic that some bacteria preferentially grow in tumor environments, achieving engineered bacterial lysis and anti‐cancer protein release in tumors based on the density of bacteria.^136^, ^137^

Bacteria as a delivery platform can release a variety of cargoes. Anti‐cancer cytokine IL‐2 can be expressed by genetically modified bacteria, promoting T‐cell proliferation and reducing tumor growth.^138^, ^139^ In mouse models, intraperitoneal infection with attenuated *S. typhimurium* showed a greatly preferential accumulation within tumor tissue. The anti‐angiogenic agent endostatin (S636/pES) carried by bacteria promotes apoptosis and reduces tumor angiogenesis.^140^ Similarly, the delivery of tissue inhibitor of metalloproteinases 2 (TIMP‐2) by attenuated *Salmonella* strains increases OS of glioma patients through the inhibition of MMPs.^141^ Furthermore, researchers have attempted to transfer siRNA into tumor cells with various delivery systems to overcome siRNA's instability in circulation.^142^ With genetically engineered bacteria, siRNA can effectively exert its gene silencing function. Genetically engineered *Salmonella* carrying HIF‐1 siRNA plasmid boosts low‐dose cisplatin's anti‐cancer effects in mice.^143^ Recently, PD‐1 siRNA‐carried *Salmonella* was reported to promote cancer cell apoptosis and immunological response.^144^ Considering the recent founding that the intratumor bacteria are more common in non‐GI cancers than previously thought, researchers can use tumor‐specific bacteria strains to construct a more effective and accurate delivery mechanism using intratumor bacteria to produce a promising anti‐cancer therapy.

## 7 CONCLUSION AND FUTURE PERSPECTIVES

Despite a long history of research on tumor‐bacteria relationships, studies of mechanisms by which bacteria colonize or influence tumors are still relatively new fields, especially for intracellular bacteria. Emerging methodological advances, especially breakthroughs in next‐generation sequencing have allowed researchers to analyze intratumor bacteria more precisely. More sophisticated sampling and processing approaches reduce contamination and bias in low microbial biomass tumor samples. Today, most tumors, even those outside the GI tract, are thought to possess a unique microbial spectrum. Comparing original tumors, normal tissues, metastatic tumors, and blood samples reveals the heterogeneity of the tumor bacterial spectrum. The discovery of intracellular bacteria in tumor cells has greatly expanded our understanding of intratumor bacteria, providing new insights into how bacteria evade antibiotics and how they affect tumors. These discoveries allow intentional interference with intratumor bacteria. Using certain combinations of antibiotics along with a deeper understanding of bacterial function may allow researchers to selectively eradicate cancer‐promoting bacteria while preserving the integrity of cancer‐suppressing bacteria. In tumor therapy, as well as for early cancer diagnosis and prognosis, genetically modified bacteria can directly kill tumor cells or stimulate antitumor immune cells by preferentially accumulating in tumor tissue. After metabolic design and engineering, bacteria can become therapeutic drug delivery platforms, notably for loading small molecules that are fragile and not suitable for injectable or oral use. Clinical trials are essential for confirming the therapeutic value found in vitro or in animal models; however, the existing evidence on the efficacy of bacterial therapy remains controversial. Recent results on intratumor and intracellular bacteria may help researchers design novel treatment trials. Taken together, there are still many pressing issues in this area that need to be addressed, which require the joint efforts of scholars and further improvement of technology. Notably, intratumor bacteria research can help us better understand tumor development and may have therapeutic transformation value, making it the next anti‐cancer research hotspot.

## AUTHOR CONTRIBUTIONS

**Zhu Liu:** Investigation (equal); writing – original draft (lead). **Lian‐Lian Hong:** Investigation (supporting); writing – review and editing (supporting). **Zhi‐Qiang Ling:** Conceptualization (lead); funding acquisition (lead); writing – review and editing (lead).

## FUNDING INFORMATION

This work was funded by National Natural Science Foundation of China (81972908, 32271238), National Health Commission Science Research Fund‐Zhejiang Provincial Health Key Science and Technology Plan Project (WKJ‐ZJ‐2117), Leading Talents in Scientific and Technological Innovation from Zhejiang Provincial Ten Thousand Talents Plan (Zhejiang Provincial CPC Committee Talents [2019]‐3), Zhejiang Province Health Leader Talent (Zjwjw2021‐40), and Major Training Personnel from Zhejiang Provincial Program for Training and Development Project for 151 Talents (Zjhrss2014‐150).

## CONFLICT OF INTEREST STATEMENT

The authors declare no competing interests.

## PATIENT CONSENT FOR PUBLICATION

Not applicable.

## ETHICS APPROVAL

This study does not involve human participants.

## Data Availability

Not applicable.
